# Validation of the Edited Tromsø Infant Faces Database (E-TIF): A study on differences in the processing of children's emotional expressions

**DOI:** 10.3758/s13428-023-02163-9

**Published:** 2023-06-27

**Authors:** Almudena Duque, Gonzalo Picado, Gloria Salgado, Alfonso Salgado, Beatriz Palacios, Covadonga Chaves

**Affiliations:** 1 Facultad de Psicología, Universidad Pontificia de Salamanca, C/ Compañía 5, 37002 Salamanca, Spain; 2 Facultad de Psicología, Universidad Complutense de Madrid, Campus de Somosaguas s/n, 28223 Pozuelo de Alarcón, Spain

**Keywords:** Validation, Emotional expressions, Faces, Children

## Abstract

Images of emotional facial expressions are often used in emotion research, which has promoted the development of different databases. However, most of these standardized sets of images do not include images from infants under 2 years of age, which is relevant for psychology research, especially for perinatal psychology. The present study aims to validate the edited version of the Tromsø Infant Faces Database (E-TIF) in a large sample of participants. The original set of 119 pictures was edited. The pictures were cropped to remove nonrelevant information, fitted in an oval window, and converted to grayscale. Four hundred and eighty participants (72.9% women) took part in the study, rating the images on five dimensions: depicted emotion, clarity, intensity, valence, and genuineness. Valence scores were useful for discriminating between positive, negative, and neutral facial expressions. Results revealed that women were more accurate at recognizing emotions in children. Regarding parental status, parents, in comparison with nonparents, rated neutral expressions as more intense and genuine. They also rated sad, angry, disgusted, and fearful faces as less negative, and happy expressions as less positive. The editing and validation of the E-TIF database offers a useful tool for basic and experimental research in psychology.

## Introduction

Processing of external stimuli is conceived as a key factor in the evolutionary success of our species (Carretié et al., 2004; Pascalis & Kelly, 2009). Cognitive studies on this topic (Öhman, 1997; Öhman et al., 2000; Sokolov, 1963) have observed that our nervous system automatically reorients its processing resources towards two kinds of important stimulation: novel stimuli (i.e., unknown or unexpected) and emotional and motivational stimuli (i.e., important for the individual, such as food, danger, partners, social hierarchy). Specifically, the processing of emotional stimuli (e.g., emotional expressions) is essential for successful social interaction (Jack & Schyns, 2015; Keltner et al., 2019), as the human processing of social behavior is based on the constant practice of attributing mental states from emotional expressions (e.g., facial, verbal) in what has been widely called Theory of Mind (Brüne & Brüne-Cohrs, 2006; Molenberghs et al., 2016). Social affective stimuli are preferentially processed by the nervous system of the human species (Eslinger et al., 2021) because the satisfaction of most human needs involves social interaction, especially in children (Brown & Brown, 2015).

Some studies found that newborns and 3-, 6-, and 9-month-old infants show attentional preference for faces over distractors (Frank et al., 2009; Johnson et al., 1991; Libertus et al., 2017), indicating the evolutionary importance of face processing in socio-cognitive and social relationship development. Indeed, problems with selecting socially relevant versus irrelevant information has been associated with worse social interactions. For instance, these problems have often been observed in individuals with autism spectrum disorder (ASD, Frazier et al., 2017; Vacas et al., 2021, 2022). 

On the other hand, correct attentional orienting and a realistic understanding of children´s facial expressions are particularly important to parenthood. Parents experience certain psychological changes, which help them better adapt to their new roles and enhance their responsiveness to children (Zhang et al., 2020). Reciprocally, infants communicate their needs and mental states mainly through vocalizations and facial expressions, which convey salient information that elicits affection and nurturing from adults (Liszkowski, 2014). A parent’s ability to accurately comment on their infant’s mental state is pivotal for the development of a secure attachment relationship and adaptive social functioning in children (Katznelson, 2014; Meins et al., 2001).

Images of emotional facial expressions are often used in emotion research. In recent years, one of the most important and frequently used facial picture sets has been the Karolinska Directed Emotional Faces database (KDEF) developed by Lundqvist et al. (1998). The KDEF includes 490 pictures showing 70 individuals (35 women and 35 men) displaying seven different emotional expressions (happy, sad, angry, fearful, disgusted, surprised, and neutral) from five different angles. The KDEF has been used in a wide range of research topics, especially in cognitive (Blanco et al., 2021; Calvo & Lundqvist, 2008; Kuehne et al., 2019) and clinical studies (Calvo & Avero, 2005; Duque & Vázquez, 2015; Sanchez et al., 2013; Unruh et al., 2020). Nevertheless, the KDEF has some limitations, such as not including emotional expressions from different age groups. For instance, this database does not include children’s pictures, which are especially relevant in research areas such as perinatal psychology. Several studies have found that, compared to pregnant women from a control group, depressed pregnant women disengage their gaze faster from children's faces with negative emotions (Pearson et al., 2010). However, this finding was not observed when negative emotional expressions from adults were displayed (Pearson et al., 2010). A recent study found that attentional biases towards infant faces are only present in expecting parents (women and men) with depressive symptoms but not in women with major depression (Bohne et al., 2021), revealing the importance of emotional self-relevant material when examining cognitive processes. However, for most of these studies, infant faces were sourced from publicly available data online due to the absence of standardized sets of emotional expressions for this purpose.

Numerous studies have found that infant stimuli, especially if they display emotional content, are prioritized in the attentional system of adults (Brosch et al., 2007; Thompson-Booth et al., 2014). This attentional preference elicits caregiving behaviors in adults which are essential for infant survival (Lorenz, 1943; Stern, 2002). Furthermore, previous research observed gender differences in the processing of children´s emotional faces. Women were faster at processing both positive and negative infant faces (Proverbio et al., 2006), showed more decoding accuracy (Proverbio et al., 2007) and rated all images as more arousing and clearer than men (Maack et al., 2017).

In addition to gender differences, some studies have addressed whether parenthood has differential effects on the processing of infant faces. For example, a review conducted by Parsons et al. (2010) reported that parents showed more arousing activity than nonparents in the reward-related mesolimbic dopamine system in response to images or videos of children. Similarly, Proverbio et al. (2006) observed that parents’ brain activity was significantly higher than that of nonparents during the processing of infant facial expressions that varied in valence and intensity.

To conduct these types of studies, several databases with facial expressions of children have been developed, for example, the Child Affective Facial Expression (CAFE) database (LoBue & Thrasher, 2015), the NIMH Child Emotional Faces Picture Set (NIMH-ChEFS) (Egger et al., 2011) or the Dartmouth Database of Children Faces (Dalrymple et al., 2013). These sets include pictures with different emotional expressions (happy, sad, angry, etc.) from children between 2 and 16 years old, but they do not contain emotional faces of infants under 2 years of age. To solve this limitation, Maack et al. (2017) created the Tromsø Infant Faces database (TIF), a standardized and freely available set containing full-color images of the emotional expressions of 18 Caucasian infants aged 4 to 12 months old. Another set of affective facial pictures of infants is the City Infant Faces Database (Webb et al., 2018), which consists of 154 facial stimuli expressed by babies aged between 0 and 12 months old. However, the use of this set is limited because the children’s parents took the pictures, and therefore, the images are too heterogeneous in terms of image quality, head position, etc.

According to some authors, to maximize the emotional salience of facial expressions, it is recommended to limit the displayed image to the facial area, removing nonrelevant information such as hair, neck, and other surrounding parts (Calvo & Lundqvist, 2008). Also, previous studies have used black and white or grayscale stimuli to reduce distracting elements such as color and brightness of the pictures (Blanco et al., 2017; Calvo et al., 2008). Following these guidelines, the main objective of this study was to edit the photographs contained in the TIF database and validate them in a large sample of participants. According to the original study (Maack et al., 2017), the images were assessed on five subjective scales: (a) depicted expression, (b) clarity, (c) intensity, (d) valence and (e) genuineness of the expression.

Additionally, our second purpose was to explore differences in the processing of infant faces between men and women, and parents and nonparents. Therefore, considering prior findings, the following was expected:

1. Compared with men, women would show more decoding accuracy (Proverbio et al., 2007).
2. Compared with men, women will rate all types of facial expressions as clearer and more intense. No differences were expected regarding valence and genuineness (Maack et al., 2017).
3. In comparison with nonparents, parents will show greater decoding accuracy due to their life experience (Proverbio et al., 2006).
4. In comparison with nonparents, parents will rate neutral and sad expressions as clearer and more intense. No differences were expected regarding valence and genuineness (Maack et al., 2017).

## Methods

### Participants

A sample of 480 participants (350 women) voluntarily took part in the study after providing informed consent. Participants who rated less than 80% of the facial expressions in each block were excluded from the data analysis. No differences were found between blocks in the number of participants excluded [*X*^2^ (3) = 2.104, *p* = .551]. Sixty-three participants were excluded from Block 1, 53 from Block 2, 55 from Block 3, and 57 from Block 4.

All participants were aged between 18 and 77 (mean = 40.88, *SD* = 14.94). Similar to prior studies (Moltó et al., 2013), the sample had a distribution ratio of women to men of 2:1. Regarding parental status, 276 participants (57.5%) reported being parents.

Sample size was previously calculated using G*Power 3.1.9.4 software for a repeated measures analysis of variance (ANOVA) within-between interactions. In an attempt to obtain a small effect size (.15), considering an alpha level of .01, four groups (women–men, parents–nonparents), seven measures (one for each type of emotional expression), expecting a statistical power of .95, and a correlation among repeated measures of .20, G*Power results indicated that a minimum sample of 196 participants would be necessary to obtain significant differences. However, since previous validation studies have used samples with over 200 participants (Grimaldos et al., 2021; Goeleven et al., 2008; Kurdi et al., 2017; Lang et al., 2008), we decided to increase the number of participants.

### Materials

**Fig. 1:**
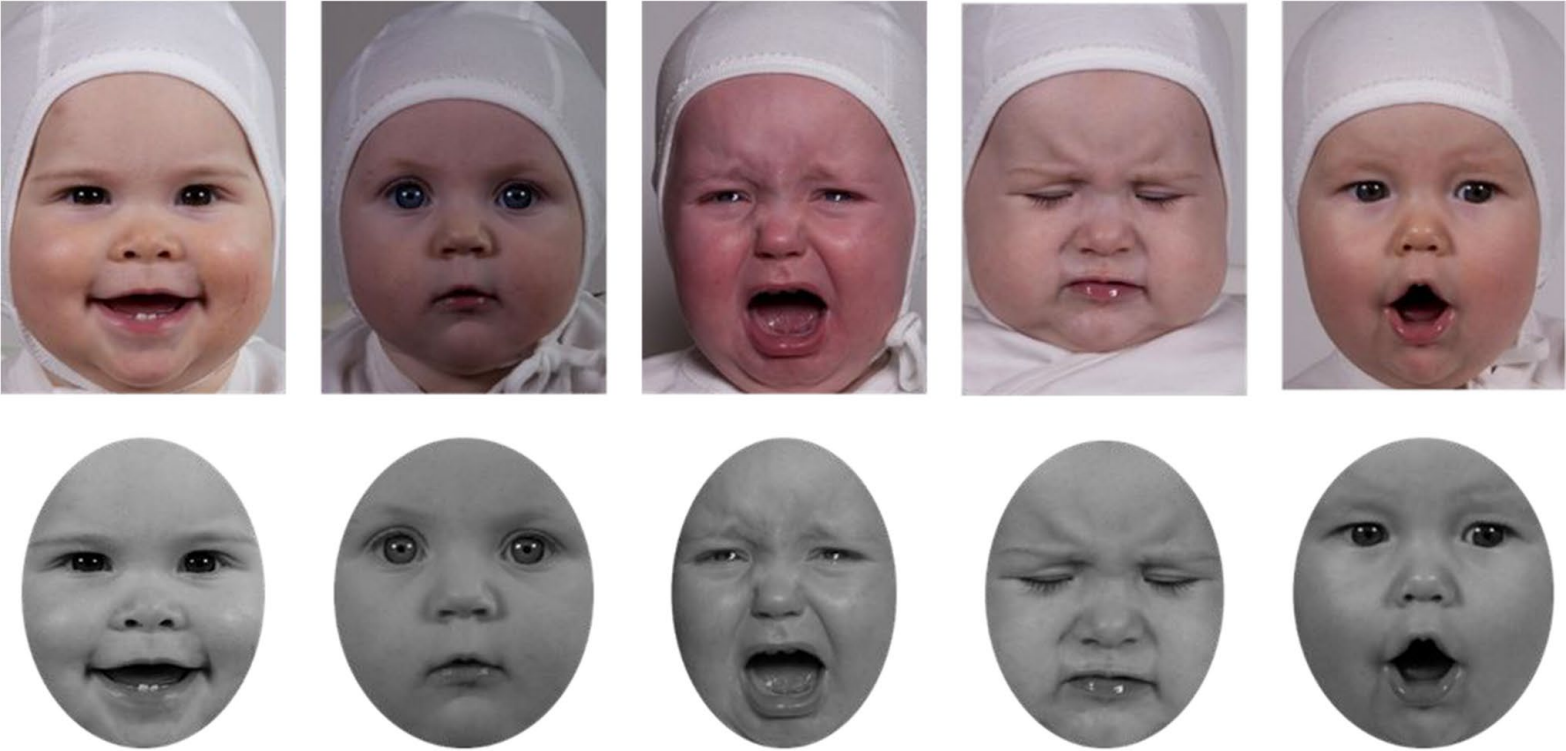
Example of the original TIF expressions and the cropped and converted to grayscale final edition

The pictures were obtained from the Tromsø Infant Faces database. We obtained permission to edit and validate the images from the corresponding author (Gerit Pfuhl) of the original version. The TIF database is composed of emotional facial expressions from 18 infants (10 girls and 8 boys) ranging in age from 4 to 12 months. For each infant there are two images of happy expressions, one of a sad expression, one of a disgusted expression, one of a neutral expression, and one depicting surprise, fear, or anger. This set of 119 original TIF pictures was edited for this study. First, following Calvo & Lundqvist (2008), the pictures were cropped to remove nonrelevant information (every child was wearing a white cap tied under her/his chin) and fitted in an oval window. Then, all images were converted to grayscale for the purpose of homogenizing visual aspects such as brightness (Fig. 1).

### Procedure

The study was set up using Qualtrics (Provo, UT, 2017) and distributed through social media. The study was launched in Spanish.

To avoid cognitive overload and fatigue, the 119 images were divided into four blocks. The first block consisted of 30 pictures and was rated by 110 participants. The second block consisted of 30 pictures and was rated by 128 participants. The third block consisted of 30 pictures and was rated by 124 participants. The fourth block consisted of 29 images and was rated by 118 participants. Images were randomly assigned to each block.

Emotion, clarity, intensity, valence, and genuineness ratings were obtained for each picture. Participants completed the survey on their computers or mobile devices. Each facial expression and the five rating scales were presented on the same screen. First, participants were asked to choose the emotion displayed by the infant *(i.e., “What emotion do you think the baby is experiencing? Happy, sad, surprised, disgusted, fearful, angry, or neutral”*). Then, clarity, intensity, valence, and genuineness were measured for each image *(i.e., “How would you rate the emotion expressed by the baby according to the following characteristics?”).* A five-point Likert scale was used, ranging from “0 = very ambiguous” to “5 = very clear” (clarity), “0 = very weak” to “5 = very strong” (intensity), “0 = very negative” to “5 = very positive” (valence), and “0 = not real” to “5 = very real” (genuineness). Each emotional expression was rated by a minimum of 109 and a maximum of 128 participants (*M* = 120, *SD* = 7.10).

The mean duration of the survey was 23 minutes. Participation was voluntary and participants did not receive any reward. The study was approved by the university ethics committee and was conducted in compliance with the Declaration of Helsinki.

Statistical analyses were performed using SPSS 25 and G*Power 3.1.9.4.

### Data analysis

Descriptive statistics (mean and standard deviation) were obtained for each picture.

To analyze differences in accuracy, clarity, intensity, valence, and genuineness, we conducted one-way ANOVAs using the different emotional expressions as independent variables. Post hoc tests (Games-Howell) were used to further analyze significant differences.

To explore differences between women and men, and parents and nonparents, linear mixed models (LMM) were conducted (estimated using maximum likelihood). LMM cope with missing data more easily than repeated-measures ANOVA. In the case of this dataset and due to the random assignment of the pictures to the different blocks, a number of participants were excluded because they did not rate facial expressions of any emotion. For this reason, an LMM approach was most appropriate.

We included accuracy, clarity, intensity, valence, and genuineness as the dependent variables. and added emotion, gender, parental status, and multiple interactions as fixed effects. We included Participant as a random effect. Age was used as a covariate. To explore significant differences, corrected Bonferroni post hoc tests were used.

## Results

### Sample characteristics

Sample characteristics are summarized in Table 1. No differences in age [*F*(3, 479) = .189; *p* = .904], gender [*X*^2^(3) = 5.958, *p* = .114], or parental status distributions [*X*^2^(3) = 1.156, *p* = .764] were found between the four blocks. However, we found differences in age regarding gender (*t*(200.582) = 2.906, *p* = .004, *g =* 2.37, 95% CI = [1.54, 8.02]) and parental status, (*t*(478) = −17.163, *p* = .000, *d* = .16, 95% CI = [−20.77, −16.50]). The mean age for women and men was 39.59 (*SD* = 14.04) and 44.37 (*SD* = 16.69) years, respectively. The mean age for parents and nonparents was 48.80 (*SD* = 10.91) and 30.17 (*SD* = 12.83) years, respectively.

**Table 1:** Sample characteristics

	Block 1 (*N*=110)	Block 2 (*N*=128)	Block 3 (*N*=124)	Block 4 (*N*=118)	Total sample (*N*=480)
Age *(SD)*	40.69 (*14.72*)	40.98 (*14.99*)	40.22 (*15.26*)	41.64 (*14.88*)	40.88 (*14.94*)
Gender (N)					
Women	90 (81.81%)	91 (71.09%)	88 (70.97%)	81 (68.64%)	350 (72.92%)
Men	20 (18.18%)	37 (28.90%)	36 (29.03%)	37 (31.35%)	130 (27.08%)
Parental status					
Parents	67 (60.91%)	75 (58.59%)	70 (56.45%)	64 (54.24%)	276 (57.5%)
Non-parents	43 (39.09%)	53 (41.41%)	54 (43.55%)	54 (45.76%)	204 (42.5%)

### Accuracy, clarity, intensity, valence, and genuineness analyses

As a means to explore differences between the original version of the TIF and the edited version (E-TIF), accuracy rates were calculated for each picture using the proportion of participants who correctly identified the original TIF target emotion. The mean accuracy rate was 59.53% (*SD* = 24.63, 95% CI = [55.05, 64.00]). The one-way ANOVA reached marginal significance [*F*(6, 112) = 2.123, *p* = .056, *η*^*2*^ = .10]; however, corrected Bonferroni post hoc tests did not show statistical differences between different emotions in accuracy (all *p*s > .10). Mean accuracy rates were 69.98% (*SD =* 26.28, 95% CI = [60.17, 79.79]) for happy expressions, 67.15% (*SD =* 26.68, 95% CI = [44.85, 89.44]) for fearful expressions, 64.25% (*SD =* 16.70, 95% CI = [51.42, 77.09]) for angry expressions, 61.18% (*SD =* 24.95, 95% CI = [46.10, 76.26]) for surprised expressions, 52.94% (*SD =* 26.16, 95% CI = [42.37, 63.51]) for neutral expressions, 52.88% (*SD =* 24.29, 95% CI = [36.57, 69.20]) for disgusted expressions, and 50.70% (*SD =* 17.68, 95% CI = [42.86, 58.53]) for sad expressions.

Additional analyses were conducted to compare ratings of clarity, intensity and valence^1^ for the overall set of images with those found in the original version. On average, images from the original version of the TIF were rated as more clear (*t* (118) = 8.780, *p* < .001, r = .62), more intense (*t* (118) = 10.941, *p* < .001, r = .71) and more positive (*t* (118) = 8.466, *p* < .001, *r* = .61) than the edited ones. Results are shown in Table 2.

**Table 2:** Comparison between the original TIF and the E-TIF

	Original TIF	E-TIF	Mean differences CI (95%)
	Mean *(SD)*	Mean *(SD)*	
Clarity	3.45 *(.49)*	3.12 *(.58)*	.33 (.25–.40)
Intensity	3.45 *(.57)*	3.11 *(.67)*	.33 (.27–.39)
Valence	2.92 *(.84)*	2.69 *(.74)*	.23 (.17–.28)

*CI* confidence interval

To explore clarity, intensity, valence and genuineness of emotional expressions, the highest-rated emotion (i.e., highest percentage of agreement) was used as the valid emotion for every edited picture. Of the original 119 images, 25 expressions were rated as happy, 17 as sad, 17 as angry, 12 as surprised, 8 as disgusted, 12 as fearful, and 25 as neutral. Three expressions were similarly rated as different emotions, so we labelled these faces as ambiguous.

A one-way ANOVA revealed that there were statistically significant differences in clarity [*F*(6, 109) = 9.489, *p* < .001, *η*^*2*^ = .34], intensity [*F*(6, 109) = 17.561, *p* < .001, *η*^*2*^ = .49], valence [*F*(6, 109) = 83.628, *p* < .001, *η*^*2*^ = .82] and genuineness [*F*(6, 109) = 5.997, *p* < .001, *η*^*2*^ = .25] between the different emotional expressions (Table 3). Post hoc analyses (Bonferroni corrected) revealed that neutral expressions were rated as less clear than happy, sad, angry, surprised, and fearful expressions (all *p*s < .009). No differences were found between neutral and disgusted expressions (*p* = .817).

**Table 3:** Means and standard deviations for clarity, intensity, valence, and genuineness for the overall sample

	Clarity	Intensity	Valence	Genuineness
	Mean *(SD)* CI (95%)	Mean *(SD)* CI (95%)	Mean *(SD)* CI (95%)	Mean *(SD)* CI (95%)
Happy	3.48 *(.63)* (3.22–3.74)	3.21 *(.59)* (2.97–3.46)	3.74 *(.40)* (3.57–3.90)	3.89 *(.34)* (3.75–4.03)
Sad	3.37 *(.41)* (3.16–3.58)	3.63 *(.44)* (3.41–3.86)	1.94 *(.23)* (1.82–2.06)	3.94 *(.24)* (3.82–4.06)
Angry	3.24 *(.54)* (2.97–3.52)	3.47 *(.65)* (3.13–3.80)	2.13 *(.32)* (1.96–2.29)	3.81 *(.28)* (3.67–3.96)
Surprised	3.23 *(.50)* (2.91–3.54)	3.16 *(.46)* (2.87–3.45)	3.16 *(.49)* (2.85–3.47)	3.83 *(.24)* (3.68–3.99)
Disgusted	2.82 *(.53)* (2.38–3.26)	3.08 *(.52)* (2.64–3.51)	2.20 *(.18)* (2.04–2.36)	3.66 *(.32)* (3.39–3.93)
Fearful	3.20 *(.43)* (2.92–3.47)	3.43 *(.51)* (3.12–3.75)	1.98 *(.24)* (1.83–2.14)	3.84 *(.27)* (3.67–4.02)
Neutral	2.56 *(.31)* (2.43–2.68)	2.29 *(.16)* (2.23–2.36)	2.83 *(.27)* (2.72–2.94)	3.52 *(.15)* (3.46–3.59)

*CI* confidence interval

Regarding intensity, neutral expressions were rated as less intense than the other emotional expressions (*p* < .035). However, no differences were found among other emotional expressions (all *p*s > .122).

Regarding valence, post hoc analyses showed that happy faces were rated as more positive than the rest of expressions (all *p*s < .028). Sad expressions were evaluated as the most negative, and statistically significant differences were observed in comparison to surprised (*p* < .001) and neutral expressions (*p* < .001). No differences were observed between sad expressions and other negative emotions like disgust (*p* = .096), anger (*p* = .446) and fear (*p* = .999). Neutral expressions were rated as more positive than sad, angry, disgusted, and fearful expressions (all *p*s < .001), but no differences were found in comparison with surprised images (*p* = .363).

Finally, neutral expressions were rated as less genuine than happy, sad, angry, surprised, and fearful expressions (all *p*s < .026). Neutral pictures did not differ from the ones depicting disgust (*p* = .894) in genuineness. No differences were shown among other emotional expressions in genuineness (all *p*s > .364).

### Gender and parental status differences

A linear mixed model (LMM) was conducted using accuracy as the dependent variable. Emotion, gender, parental status, and multiple interactions were added as fixed effects. A significant main effect of gender was found [*F*(1, 463.185) = 5.368, *p* = .021, *η*^*2*^ = .011], showing that women were more accurate than men at identifying emotions across all types of expressions (Table 4). Mean accuracy ratings for women and men were 52.4% (*SD* = .70, 95% CI = [51.10, 53.70]) and 49.5% (*SD* = 1.10, 95% CI = [47.40, 51.60]), respectively. A marginally significant parental status × emotion interaction effect was found [*F*(6, 460.490) = 2.088, *p* = .053, *η*^*2*^ = .026]. Corrected Bonferroni post hoc tests showed that parents tended to be more accurate than nonparents at identifying neutral expressions (*p* =.004, Table 5). No interaction effects between gender and emotion [*F*(6, 460.494) = 1.248, *p* = .281, *η*^*2*^ = .016] or gender × parental status × emotion [*F*(6, 460.492) = .470, *p* = .830, *η*^*2*^ = .006] were found.

**Table 4:** Means and standard deviations for accuracy, clarity, intensity, valence, and genuineness, broken down by gender

	Accuracy		Clarity		Intensity		Valence		Genuineness	
	Women	Men	Women	Men	Women	Men	Women	Men	Women	Men
	Mean *(SD)* CI (95%)	Mean *(SD)* CI (95%)	Mean *(SD)* CI (95%)	Mean *(SD)* CI (95%)	Mean *(SD)* CI (95%)	Mean *(SD)* CI (95%)	Mean *(SD)* CI (95%)	Mean *(SD)* CI (95%)	Mean *(SD)* CI (95%)	Mean *(SD)* CI (95%)
Happy	68.5% *(1.00)* (66.60–70.50)	68.9% *(1.60)* (65.80–72.10)	3.53 *(.04)* (3.45–3.61)	3.46 *(.06)* (3.33–3.58)	3.21 *(.04)* (3.14–3.28)	3.25 *(.06)* (3.14–3.37)	3.82 *(.03)* (3.76–3.88)	3.69 *(.05)* (3.59–3.79)	3.97 *(.03)* (3.90–4.04)	3.80 *(.06)* (3.69–3.91)
Sad	47.6% *(1.30)* (45.10–50.20)	46.3% *(2.20)* (42.10–50.50)	3.39 *(.04)* (3.30–3.47)	3.30 *(.07)* (3.16–3.44)	3.70 *(.03)* (3.63–3.77)	3.48 *(.06)* (3.37–3.60)	1.91 *(0.5)* (1.80–2.02)	1.92 *(.09)* (1.74–2.10)	4.01 *(.04)* (3.93–4.08)	3.72 *(.06)* (3.60–3.84)
Angry	55.3% *(2.30)* (50.80–59.80)	46.6% *(3.80)* (39.20–54.00)	3.22 *(.04)* (3.13–3.30)	3.39 *(.07)* (3.25–3.54)	3.48 *(.03)* (3.41–3.55)	3.53 *(.06)* (3.41–3.64)	2.07 *(.05)* (1.97–2.17)	2.16 *(.08)* (2.00–2.32)	3.84 *(.04)* (3.77–3.92)	3.73 *(.06)* (3.60–3.85)
Surprised	51.2% *(1.60)* (48.10–54.30)	48.7% *(2.60)* (43.60–53.80)	3.31 *(.05)* (3.21–3.42)	3.08 *(.09)* (2.90–3.25)	3.12 *(.05)* (3.02–3.22)	3.01 *(.08)* (2.85–3.18)	3.23 *(.04)* (3.16–3.30)	3.14 *(.06)* (3.02–3.26)	3.89 *(.04)* (3.80–3.98)	3.58 *(.07)* (3.44–3.72)
Disgusted	45.3% *(1.40)* (42.50–48.10)	38.4% *(2.30)* (33.80–42.90)	3.02 *(.05)* (2.92–3.13)	2.71 *(.09)* (2.54–2.89)	3.20 *(.04)* (3.11–3.28)	3.02 *(.08)* (2.88–3.17)	2.16 *(.05)* (2.07–2.26)	2.19 *(.08)* (2.04–2.35)	3.74 *(.04)* (3.66–3.83)	3.48 *(.07)* (3.34–3.62)
Fearful	44.9% *(2.20)* (40.60–49.20)	44.5% *(3.40)* (37.80–51.20)	3.18 *(.05)* (30.8–3.28)	3.25 *(.08)* (3.09–3.41)	3.39 *(.04)* (3.30–3.48)	3.32 *(.07)* (3.17–3.47)	2.09 *(.05)* (2.00–2.19)	2.29 *(.08)* (2.14–2.44)	3.86 *(.07)* (3.77–3.94)	3.71 *(.07)* (3.58–3.84)
Neutral	54.3% *(1.30)* (51.80–56.70)	53.3% *(2.10)* (49.20–57.30)	2.55 *(.05)* (2.46–2.64)	2.57 *(.08)* (2.42–2.72)	2.25 *(.04)* (2.13–2.33)	2.38 *(.07)* (2.25–2.52)	2.87 *(.02)* (2.82–2.92)	2.80 *(.04)* (2.72–2.88)	3.57 *(.04)* (3.49–3.64)	3.38 *(.06)* (3.25– 3.50)

*CI* confidence interval

**Table 5:** Means and standard deviations for accuracy, clarity, intensity, valence and genuineness broken down by parental status

	Accuracy		Clarity		Intensity		Valence		Genuineness	
	Parents	Nonparents	Parents	Nonparents	Parents	Nonparents	Parents	Nonparents	Parents	Nonparents
	Mean *(SD)* CI (95%)	Mean *(SD)* CI (95%)	Mean *(SD)* CI (95%)	Mean *(SD)* CI (95%)	Mean *(SD)* CI (95%)	Mean *(SD)* CI (95%)	Mean *(SD)* CI (95%)	Mean *(SD)* CI (95%)	Mean *(SD)* CI (95%)	Mean *(SD)* CI (95%)
Happy	69.1% *(1.30)* (66.60–71.60)	68.4% *(1.50)* (65.40–71.30)	3.40 *(.06)* (3.39–3.60)	3.49 *(.06)* (3.37–3.61)	3.24 *(.05)* (3.15–3.34)	3.22 *(.06)* (3.11–3.33)	3.65 *(.04)* (3.57–3.73)	3.86 *(.05)* (3.77–3.95)	3.92 *(.05)* (3.82–4.01)	3.85 *(.05)* (3.75–3.96)
Sad	46.2% *(1.70)* (42.90–49.50)	47.7% *(2.00)* (43.80–51.50)	3.40 *(.06)* (3.28– 3.51)	3.28 *(.07)* (3.15–3.42)	3.56 *(.05)* (3.47–3.65)	3.62 *(.05)* (3.51–3.73)	2.11 *(.07)* (1.97–2.25)	1.72 *(.08)* (1.56–1.88)	3.94 *(.05)* (3.84–4.04)	3.79 *(.06)* (3.67–3.90)
Angry	54.6% *(2.90)* (49.00–60.20)	47.3% *(3.40)* (40.60–53.90)	3.23 *(.06)* (3.11–3.34)	3.38 *(.07)* (3.25–3.52)	3.39 *(.05)* (3.29–3.48)	3.63 *(.05)* (3.51–3.73)	2.30 *(.06)* (2.17–2.42)	1.94 *(.07)* (1.79–2.08)	3.79 *(.05)* (3.69–3.89)	3.79 *(.06)* (3.68–3.90)
Surprised	51.5% *(2.00)* (47.60–55.40)	48.4% *(2.30)* (43.80–53.00)	3.11 *(.07)* (2.97–3.25)	3.28 *(.08)* (3.11–3.44)	3.01 *(.07)* (2.89–3.14)	3.12 *(.08)* (2.97–3.27)	3.18 *(.05)* (3.09–3.27)	3.19 *(.05)* (3.08–3.30)	3.78 *(.06)* (3.66–3.89)	3.69 *(.07)* (3.56–3.83)
Disgusted	43.2% *(1.80)* (38.00–48.50)	40.4% *(2.10)* (36.20–44.60)	2.89 *(.07)* (2.75–3.02)	2.85 *(.08)* (2.69–3.01)	3.03 *(.06)* (2.92–3.14)	3.19 *(.07)* (3.05–3.32)	2.30 *(.06)* (2.18–2.43)	2.05 *(.08)* (1.91–2.20)	3.63 *(.06)* (3.52–3.75)	3.59 *(.07)* (3.45–3.72)
Fearful	43.2% *(2.70)* (38.00–48.50)	46.2% *(3.10)* (40.10–52.30)	3.18 *(.06)* (3.05–3.31)	3.25 *(.07)* (3.10–3.39)	3.27 *(.06)* (3.15–3.39)	3.44 *(.07)* (3.30–3.58)	2.31 *(.06)* (2.19– 2.43)	2.07 *(.07)* (1.93–2.21)	3.80 *(.06)* (3.69–3.90)	3.77 *(.06)* (3.64–3.90)
Neutral	51.5% *(2.00)* (47.60–55.40)	48.4% *(2.30)* (43.80–53.00)	2.65 *(.06)* (2.53–2.77)	2.47 *(.07)* (2.33–2.61)	2.43 *(.06)* (2.32–2.54)	2.20 *(.07)* (2.07–2.33)	2.91 *(.03)* (2.85–2.98)	2.76 *(.04)* (2.68–2.84)	3.58 *(.05)* (3.48–3.68)	3.36 *(.06)* (3.24– 3.48)

*CI* confidence interval

The LMM for clarity showed a significant interaction between gender and emotion [*F*(6, 466.883) = 4.767, *p* < .001, *η*^*2*^ = .057]. Post hoc analyses (Table 4) revealed that women rated disgusted (*p* = .003) and surprised expressions (*p* = .024) as clearer, while men rated expressions of anger (*p* = .040) as clearer. Happy, sad, fearful and neutral expressions were similarly rated in clarity by women and men (all *p*s >.30).

The interaction parental status × emotion also reached significance [*F*(6, 466.883) = 3.359, *p* = .003, *η*^*2*^ = .041]; however, corrected Bonferroni post hoc tests did not show statistical differences between parents and nonparents in clarity (all *p*s > .067). The gender × parental status × emotion three-way interaction was not significant [*F*(6, 466.883) = .982, *p* = .437, *η*^*2*^ = .005].

Regarding intensity, the interaction between gender and emotion from the LMM was significant [*F*(6, 479.162) = 3.447, *p* = .002, *η*^*2*^ = .041]. We conducted corrected Bonferroni post hoc tests and we observed that women evaluated sad (*p* =.001) and disgusted expressions (*p* =.040) as more intense in comparison with men (Table 4). Moreover, the interaction between parental status and emotion was also significant [*F*(6, 479.162) = 5.885, *p* < .001, *η*^*2*^ = .068]. Corrected Bonferroni post hoc tests (Table 5) showed that parents, in comparison with nonparents, rated neutral expressions as more intense (*p* =.011) and angry faces as less intense (*p* =.003). The gender × parental status × emotion three-way interaction was not significant [*F*(6, 479.162) = 1.019, *p* = .412, *η*^*2*^ = .012].

Regarding valence, the LMM showed a significant gender × emotion interaction [*F*(6, 455.662) = 2.699, *p* = .014, *η*^*2*^ = .034]. After the Bonferroni tests, we found that women rated happy expressions more positively (*p =* .030) and fearful faces more negatively (*p =* .029) in comparison with men (Table 4). Moreover, a significant parental status × emotion interaction was found, [*F*(6, 455.662) = 5.694, *p* < .001, *η*^*2*^ = .070]. Post hoc analyses showed that parents, in comparison with nonparents (Table 5), rated sad, angry, disgusted, fearful and neutral expressions less negatively (all *p*s <.013) and happy expressions less positively (*p =* .001). No differences were found in surprised faces (*p =* .917). No significant gender × parental status × emotion three-way interaction was found [*F*(6, 455.662) = .869, *p* = .517, *η*^*2*^ = .011].

The LMM for genuineness showed a significant main effect for gender [*F*(1, 480.075) = 12.453, *p* < .001, *η*^*2*^ = .025]. Specifically, women rated all types of expressions as more genuine in comparison with men (Table 4). Mean genuineness ratings for women and men were 3.84 (*SD =* .03, 95% CI = [3.78, 3.90]) and 3.63 (*SD =* .05, 95% CI = [3.53, 3.73]), respectively. The gender × emotion interaction was not significant [*F*(6, 469.849) = 1.769, *p* = .104, *η*^*2*^ = .022]. On the other hand, the interaction parental status × emotion reached significance [*F*(6, 469.849) = 2.679, *p* = .014, *η*^*2*^ = .033]. Corrected Bonferroni tests (Table 5) showed that parents rated neutral expressions as more genuine (*p* =.011) in comparison with nonparents. The gender × parental status × emotion three-way interaction was not significant [*F*(6, 469.849) = .745, *p* = .613, *η*^*2*^ = .009].

## Discussion

The main objective of the present research was to edit and validate the E-TIF database in a large sample of participants. We edited and validated a total of 119 infant pictures to make them suitable for experimental research, especially in perinatal psychology. Images were edited, removing nonrelevant information, and converted to grayscale following the guidelines used in previous studies (Calvo & Lundqvist, 2008). Then, each infant face was assessed according to five dimensions: depicted emotion, clarity, intensity, valence, and genuineness. Overall, results showed that neutral expressions were less clear, intense, and genuine than emotional expressions. These results are consistent with the original version of the TIF (Maack et al., 2017). In addition, valence scores were useful to discriminate between positive, negative, and neutral facial expressions. Therefore, the E-TIF database offers a valid resource for future emotion and cognitive research.

Our second purpose was to explore differences in the processing of infant faces between men and women, and parents and nonparents. Regarding these objectives, our first hypothesis (i.e., we expected that women would show more decoding accuracy across all types of expressions) was confirmed. We found a main effect of gender that revealed that women were more accurate at recognizing emotions in children.

Regarding clarity, our second hypothesis was only partially supported. Women rated disgusted and surprised expressions as clearer compared to men. These results are in line with previous studies that found that women are better at recognizing emotions of disgust and surprise (Baptista Menezes et al., 2017), perhaps because both emotions are classified by some authors as ambiguous (Palermo & Coltheart, 2004; Pochedly et al., 2012). Similar results were obtained regarding intensity. Women rated emotions of sadness and disgust as more intense. However, when exploring valence and authenticity, unexpected results were observed; women rated happy expressions more positively, fearful faces more negatively, and all types of expressions as more genuine compared to men.

This female advantage in the processing of emotional facial expressions of infants has been explained through some evolutionary hypotheses as the “primary caretaker hypothesis” (Babchuk et al., 1985; Baptista Menezes et al., 2017; Hampson et al., 2006; Parsons et al., 2021). These authors hypothesized that gender differences in identifying infant emotional expressions are related to selective pressures pertinent to the caretaking role. That is, the gender that has predominantly taken the responsibility of infant caretaking through evolutionary time showed better skills that are important for the survival of offspring, regardless of prior personal caretaking experience. In most mammalian animals, this function falls on females (Trivers, 1972). On the other hand, some authors argue that these differences could be explained by gender-typed socialization experiences, where women are provided more opportunities to assume caretaking roles (Hall, 1984; Henley, 1977; Weitz, 1974).

Our third hypothesis was not confirmed. We did not observe a general greater accuracy in the recognition of emotions in the parent´s group. Although this result was unexpected, it is in line with the original version of the TIF (Maack et al., 2017). Previous studies found that there is a familiarity effect on the accuracy of emotion recognition, i.e., familiar faces are more likely to be correctly identified (Huynh et al., 2010; Li et al., 2019; Liccione et al., 2014). Since the TIF stimuli used in our study was unfamiliar to our participants, unfamiliarity could be used to explain why parents did not show greater accuracy at emotion recognition in comparison with nonparents. Considering these findings, it could be hypothesized that the parents’ advantage would be visible only when the stimuli used for emotion recognition were from their descendants (Ranote et al., 2004; Swain et al., 2007).

Our fourth hypothesis was only partially confirmed. We found that parents rated neutral expressions as more intense, but not sad expressions, as we hypothesized. Some authors suggest that parents’ range of intensity may be wider because they are frequently exposed to the intense emotions of their children. As a consequence, they are likely to assign lower intensity to infant emotional faces (Arteche et al., 2016). Our results were consistent with the idea of overexposure. We also found that parents, in comparison with nonparents, rated neutral expressions as more genuine. They also rated sad, angry, disgusted, and fearful faces as less negative, and happy expressions as less positive.

The edited E-TIF database has some strengths but also some limitations. Regarding strengths, we believe that the edition of the images (i.e., removing nonrelevant information and converting to grayscale) is very relevant for studies that assess attentional biases in facial emotions because nonrelevant information (e.g., hair, ears, etc.) and color variations can affect visual attentional deployment. Moreover, this edition allows researchers to use these stimuli in combination with others from similar databases, for example, the edited KDEF (Goeleven et al., 2008; Sanchez & Vazquez, 2013). Finally, a key strength of the present study was the large sample used to validate this edited version of the E-TIF. One of the most important limitations in the validation of emotional facial expressions is the low participation of men (Goeleven et al., 2008; Sanchez & Vazquez, 2013; Webb et al., 2018), which limits the use of stimuli in this population. In our study, the sample had a distribution ratio of women to men of 2:1, not unlike previous large studies on image validation (Grimaldos et al., 2021; Moltó et al., 2013). Moreover, the size of the sample allowed for the validation of infant facial expressions on parents, unlike previous studies that created and validated similar stimuli (Webb, et al., 2018).

Some limitations need to be acknowledged. First, the edited E-TIF pictures were rated as less clear, intense, and positive than the original ones. This finding could be explained by the modifications performed on the images, suggesting that the removal of nonrelevant information and the conversion to grayscale could decrease the clarity and intensity of expressions. Nevertheless, this result could be also explained by cross-cultural differences between the Norwegian and Spanish sample. In fact, cultural variation in emotion intensity perception has been well documented (Ekman et al., 1987; Engelmann & Pogosyan, 2013; Matsumoto, 1990).

Second, the physical characteristics of the stimuli (e.g., size, resolution, and brightness) were not consistent between participants due to the survey being completed on their own computers or mobile devices. While online studies facilitate participant recruitment, they present the main limitation of lack of control over the conditions in which the study is carried out. Nevertheless, during the last 2 years in which travel restrictions and lockdowns were frequent, information and communications technology (ICT) were the only tools available for research. On the other hand, some authors consider that the use of ICT tools (mainly smartphones) for psychological research has some advantages such as a more naturalistic approach (Wang & He, 2015).

The third limitation is that the TIF database only contains Caucasian infants, which could limit its use on non-Caucasian samples. Although there are some databases with images of infants from different ethnicities (Cheng et al., 2015), none of them include images of infants under 2 years of age from different ethnicities. Further research is necessary to develop databases that are able to close this gap.

Despite these limitations, the edition and validation of the E-TIF database offers a useful tool for basic and experimental research in psychology. Although these validated and edited images can be used in studies with different samples, perinatal psychology studies could benefit greatly from this resource. Available studies in this field (e.g., attentional biases in perinatal depression) have used stimuli (i.e., infant faces) collected from public sources online due to the absence of standardized sets of emotional expressions for this purpose. We hope our validated edition can help researchers conduct future empirical studies to improve knowledge on perinatal depression, as well as available psychological treatments.

## Data Availability

The datasets generated during the current study are available on the OSF repository, https://osf.io/qm8zy/?view_only=44cd89982d6944acada30e6ae8a8eb9d. However, the edited images of the E-TIF database are not publicly available during the current study because they contain sensitive material (image of infants). They are available upon request for research purposes by e-mailing aduquesa@upsa.es.

## Footnotes

1 Genuineness ratings were not reported in the original version of the TIF.
